# Asymptomatic female softball pitchers have altered hip morphology and cartilage composition

**DOI:** 10.1038/s41598-025-87839-2

**Published:** 2025-01-25

**Authors:** Stuart J. Warden, Sally L. Coburn, Robyn K. Fuchs, Rachel K. Surowiec, Julio Carballido-Gamio, Joanne L. Kemp, Peter K. Jalaie, David F. Hanff, Antony J. R. Palmer, Scott J. Fernquest, Kay M. Crossley, Joshua J. Heerey

**Affiliations:** 1 Department of Physical Therapy, School of Health and Human Sciences, Indiana University Indianapolis, 1050 Wishard Blvd, RG-3147, Indianapolis, IN 46202 USA; 2 La Trobe Sport & Exercise Medicine Research Centre, School of Allied Health, Human Services and Sport, La Trobe University, Melbourne, VIC Australia; 3 Tom and Julia Wood College of Osteopathic Medicine, Marian University, Indianapolis, IN USA; 4 Weldon School of Biomedical Engineering, Purdue University, West Lafayette, IN USA; 5 Department of Radiology, School of Medicine, University of Colorado Anschutz Medical Campus, Aurora, CO USA; 6 Department of Radiology and Nuclear Medicine, Erasmus University Medical Centre, Rotterdam, The Netherlands; 7 Nuffield Department of Orthopaedics, Rheumatology and Musculoskeletal Sciences, University of Oxford, Oxford, UK

**Keywords:** Arthritis, Cam morphology, Femoroacetabular impingement, Hip joint, Magnetic resonance imaging, Osteoarthritis, Musculoskeletal system, Orthopaedics

## Abstract

Few studies have explored hip morphology and cartilage composition in female athletes or the impact of asymmetric repetitive loading, such as occurs during softball pitching. The current cross-sectional study assessed bilateral bony hip morphology on computed tomography imaging in collegiate-level softball pitchers (‘Pitch1’, *n* = 25) and cross-country runners (‘Run’, *n* = 13). Magnetic resonance imaging was used to assess cartilage relaxation times in a second cohort of pitchers (‘Pitch2’, *n* = 10) and non-athletic controls (‘Con’, *n* = 4). Pitch1 had 52% greater maximum alpha angle than Run (*p* < 0.001) and were 21.3 (95% CI 2.4 to 192.0) times more likely to have an alpha angle ≥ 60° within at least one hip. Pitch2 had longer T2 relaxation times in the superior femoral cartilage of the drive leg (same side as the throwing arm) and stride leg than Con (all *p* < 0.02). The drive leg in Pitch2 had longer T1ρ and T2 relaxation times in the superior femoral cartilage compared to the stride leg (all *p* ≤ 0.03). Asymptomatic softball pitchers exhibit altered bony hip morphology and cartilage composition compared to cross-country runners and non-athletic controls, respectively. They also exhibit asymmetry in cartilage composition. Further studies with larger sample sizes are warranted and any potential long-term consequences of the changes in terms of symptom and osteoarthritis development requires investigation.

**Supplementary Information:**

The online version contains supplementary material available at 10.1038/s41598-025-87839-2.

## Introduction

The impact of physical activity on joints and cartilage remains poorly understood^1^. At the hip, cartilage health appears to be influenced by an interaction between mechanical loading and joint morphology^2,3^. Common morphological variants include cam morphology (femoral head asphericity) and pincer morphology (acetabular overcoverage), with cam morphology being twice as common in athletes than the general population supporting a role of mechanical loading in its formation^4^. The morphological variants are often asymptomatic^5,6^; however, they represent risk factors for femoroacetabular impingement (FAI) syndrome^7^—a motion-related disorder where symptoms may be caused by impact between the femoral head-neck junction and acetabular rim^8^. FAI syndrome is associated with acetabular labral tears, chondral damage, and osteoarthritis (OA) development^3,9–12^.

The prevalence of bony hip morphology has been explored in a variety of athletic populations^6^. However, studies have predominantly involved males and athletes competing in a limited number of high impact sports, such as football/soccer and ice hockey. Studies in male populations may not be translatable to female cohorts. Further data are required in females as: (1) females are physiologically, anatomically and biomechanically different than males^13^, (2) hip morphology is different between sexes and sex-related differences in FAI syndrome have been reported^14,15^, (3) the burden of hip OA and subsequent joint replacement surgery is higher in females than in males^16^, and (4) females continue to be underrepresented within sport and exercise science research^17^.

Coupled with the limited number of studies exploring bony hip morphology in female athletes, few studies have explored the impact of asymmetrical loading. Study of individuals exposed to asymmetrical loading enables within-subject assessment of the interaction between mechanical loading and bony hip morphology while controlling for the impact of inherited or other systemic factors. Softball pitchers represent a unique population in this sense. Softball is predominantly a female sport and, due to a lack of pitch count restrictions, a softball pitcher can throw in excess of 1,000 pitches during a three-day tournament^18^. The repetitive loading during pitching is asymmetrical with the stride leg (i.e., leg opposite the pitching arm) exposed to 35% greater vertical ground reaction forces during pitching than the contralateral drive leg^19^. The asymmetrical loading induces differential adaptation with the proximal femur in the stride leg having greater bone density and strength than in the drive leg^20,21^. To our knowledge, only one previous study has reported on the prevalence of cam morphology in the bilateral hips of individuals participating in a sport potentially with asymmetrical loading. Dickenson et al.^22^ reported a higher prevalence of cam morphology in the trail legs of elite golf players; however, the overall prevalence of cam morphology was low (8.2%; 9-out-of-110 hips) and the study was limited to males.

In addition to variations in bony hip morphology, early changes in cartilage composition can be assessed prior to radiographic OA changes^23^. Identifying pre-morphological cartilage changes may prove vital for both predicting progression and implementing disease modifying interventions. T1ρ relaxation time mapping using magnetic resonance imaging (MRI) provides a surrogate indicator of cartilage proteoglycan content, whereas T2 relaxation time mapping reflects water and collagen content^24–26^. T1ρ and T2 values are both elevated in mild-to-moderate hip OA^27^, representing decreased proteoglycan content and increased water content, respectively^28,29^. Similar observations have been observed in FAI syndrome^30,31^.

The aims of the current study were to assess bone morphology and hip cartilage composition in both hips of asymptomatic female college-level softball pitchers. Our hypotheses were that softball pitchers would have: (1) different bony hip morphology than controls (female cross-country runners), (2) altered cartilage composition compared to controls (non-athletic females), and (3) asymmetry between limbs in the measured outcomes. We also compared MRI-derived volume of the gluteus maximus, medius, and minimus muscles in both legs of softball pitchers and controls (non-athletic females) as an indicator of differential loading.

## Materials and methods

### Study design

A cross-sectional study (Level of evidence, 3) was conducted in two female cohorts. In the first cohort, bony hip morphology was evaluated on computed tomography (CT) imaging previously obtained from convenience samples of collegiate-level softball pitchers (‘Pitch1’ group) and cross-country runners (‘Run’ group). The second cohort was prospectively recruited, and MRI relaxation times measured within the femoral and acetabular cartilage at the hip in convenience samples of collegiate-level softball pitchers (‘Pitch2’ group) and non-athletic controls (‘Con’ group). All procedures were approved by the Institutional Review Board and Machine Produced Radiation Safety Committee of Indiana University, and all research was performed in accordance with relevant guidelines/regulations. All participants provided written informed consent.

### Participants

Participants were included if they were aged 18–25 years and did not have a history of: (1) hip disease, injury, or surgery; (2) hip pain in the past 2 years; (3) femoral fracture or bone stress injury; or (4) exposure to lower extremity immobilization for more than 2 weeks within the past 2 years. Pitchers were included if they were competing as a pitcher in softball at the Division I, II or III level within the National Collegiate Athletic Association. Runners were included if they were competing in cross-country at the Division I, II or III level within the National Collegiate Athletic Association and did not have a history of playing in a multi-directional sport (e.g., basketball, soccer, gymnastics) more than twice per month for ≥ 4 months per year for more than 2 years. Pitchers and runners were identified from published rosters of local college teams and were recruited via email. Controls were college students from our research laboratory who self-reported they did not currently perform physical activity over-and-above activities of daily living and did not have any history of playing in a multi-directional sport.

Height and weight were measured without shoes using a calibrated stadiometer (Seca 264; Seca GmbH & Co., Hamburg, Germany) and scale (MS140-300; Brecknell, Fairmont, MN), respectively. The leg on the same side as the pitching arm (Pitch1 and 2) or preferred throwing arm (Run and Con) was defined as the drive leg (‘Drive’ group). The contralateral leg was defined as the stride leg (‘Stride’ group). Areal bone mineral density (aBMD) of the total hip of each leg was measured by dual-energy x-ray absorptiometry using the manufacturers’ recommended protocol (Norland Elite; Norland at Swissray, Fort Atkinson, WI).

### Computed tomography

CT scans previously acquired as part of a study exploring asymmetry of proximal femur bone health in 25 softball pitchers (Pitch1), and 13 runners (Run) were analyzed^20^. The previous study acquired scans in 15 runners; however, two runners were excluded from the current analyses because of a prolonged history of competing in a multi-directional sport (i.e., soccer) associated with altered bony hip morphology.

Scans were obtained using a multislice CT scanner (Biograph128 mCT; Siemens Healthcare, Knoxville, TN) operating at 120 kVp, 320 mAs, 128 × 0.6 collimation, and pitch 0.8. The scan region spanned from 1 cm superior to the acetabulum to 5 cm distal to the lesser trochanter. Images were axially reconstructed at 1.0 mm slice thickness using a B60s convolution kernel, 512 × 512 matrix, and reconstruction diameter of 50 cm (reconstructed voxel size = 0.976 × 0.976 × 1.0 mm^3^).

**Fig. 1:**
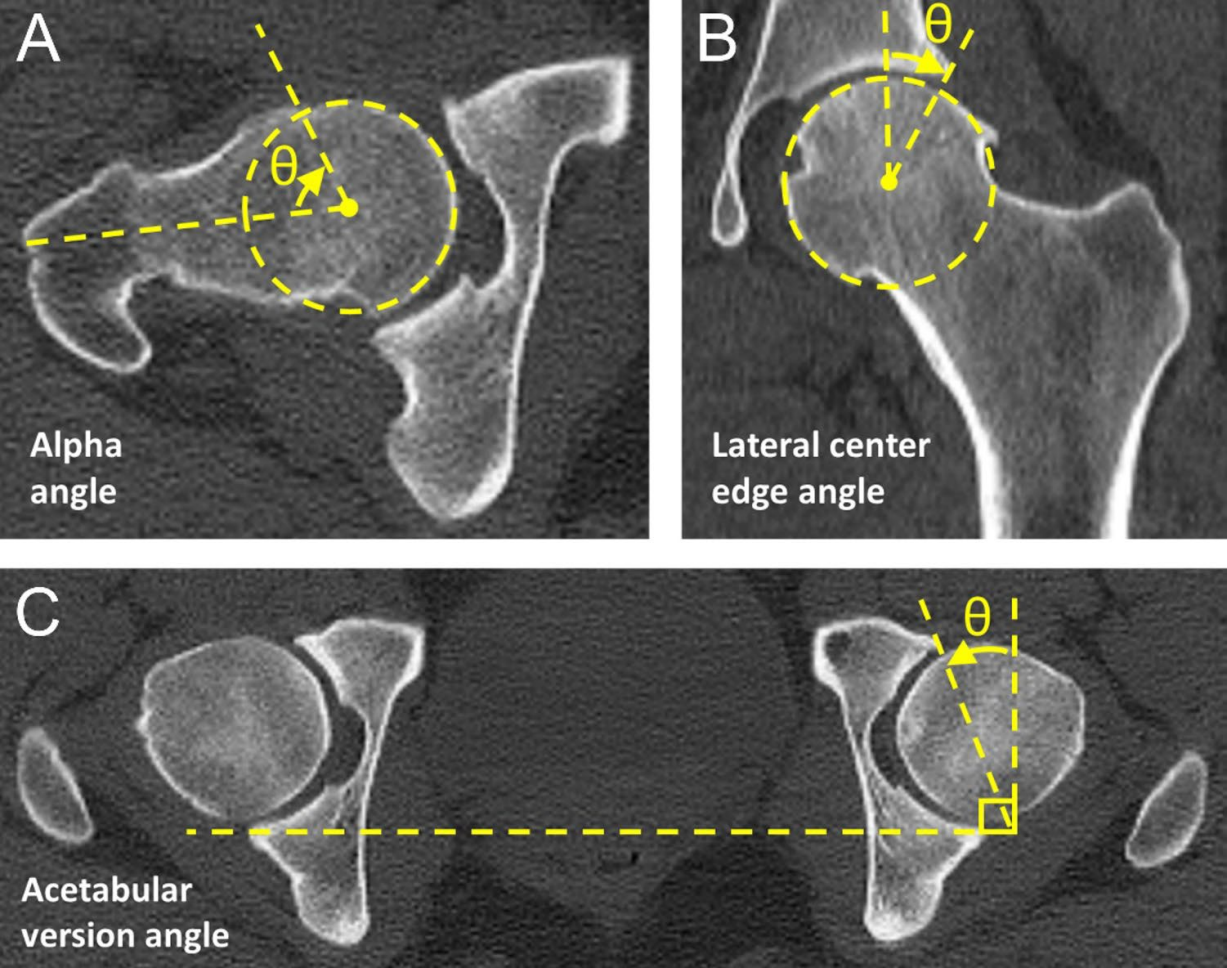
Assessment of morphometry on CT scans. (**A**) Alpha angle was measured between a line connecting the centers of the femoral head and its neck and a line from the center of the femoral head to where the distance from the center of the head exceeded its radius. (**B**) Lateral center edge angle was measured as the angle between the vertical axis of the pelvis and a line connecting the center of the femoral head and lateral acetabular margin. (**C**) Acetabular version was measured by a line drawn joining the posterior corners of the bilateral acetabulum and tangential lines drawn from the posterior corner to provide a true sagittal line for each acetabulum. The angle between the sagittal line and a line connecting the anterior and posterior corners of the acetabulum was recorded as acetabular version angle.

CT scans were analyzed using computer modeling software allowing for multiplanar 3D reconstructions (OsiriX Software V.6.0.2, Pixmeo, Geneva, Switzerland). Radial images were sampled around the axis of the femoral neck at 30° intervals. The coronal axis (12 o’clock position) was positioned parallel to the axis of the proximal femur diaphysis. The alpha angle (Fig. 1A) was measured on radial slices at the 12, 1, 2, and 3 o’clock positions to indicate femoral head asphericity (cam morphology). Presence of a cam morphology was defined as an alpha angle of ≥ 60°^32^. Lateral center-edge angle (LCEA) (Fig. 1B) and acetabular version (Fig. 1C) were measured to indicate the presence of a pincer morphology (acetabular overcoverage) and acetabular dysplasia. Acetabular dysplasia and pincer morphology were defined by a LCEA ≤ 20° and ≥ 40°, respectively^33^. Version angles of ≤ 10° and ≥ 20° were chosen to indicate acetabular retroversion and anteversion, respectively^34^.

### Magnetic resonance imaging

MRI was performed on both hips in 10 softball pitchers (Pitch2) and 4 non-athletic control individuals (Con). Participants were transported to the imaging facility via wheelchair and were non-weightbearing for 30 min prior to imaging to reduce the effects of recent loading on cartilage relaxation times^1^. We have previously shown hip joint T2 relaxation times do not change with unloading beyond 25 min^35^. MR imaging was performed on a 3T Siemens Prisma FIT MR scanner (Siemens Medical Solutions, Erlangen, Germany) with the participant in a supine position and a surface coil placed over the hip being imaged. The legs were positioned in neutral abduction/adduction and rotation with the patella facing the ceiling. Elastic straps and cushions were used to facilitate comfort and minimize movement artifacts.

The MRI protocol included the following sequences which covered the acetabulum and femoral head entirely with the center of the field of view approximating the center of the femoral head: (1) sagittal proton density (PD) weighted, (2) sagittal, coronal, and axial PD spectral attenuated inversion recovery (SPAIR), (3) sagittal T1ρ mapping, and (4) sagittal multi-echo T2 mapping (Supplementary File 1). The leg imaged first (Drive vs. Stride) was alternated between successive participants to reduce testing bias. A 15-minute interval was provided at the completion of scanning the first leg to minimize the specific absorption rate. Participants remained non-weightbearing before the opposite leg was scanned. In addition to the above sequences, an axial Dixon sequence was performed including from above the iliac crest to below the lesser trochanters (Supplementary File 1).

Images in Pitch2 obtained using SPAIR sequences were assessed using the Scoring Hip Osteoarthritis with MRI (SHOMRI) method^36^ by a musculoskeletal radiologist (DFH) with 10 years of experience. The primary features of interest were the articular cartilage (graded 0 = no loss, 1 = partial thickness loss, 2 = full thickness loss) and labrum (graded 0 = normal, 1 = abnormal signal and/or fraying, 2 = simple tear, 3 = labrocartilage separation, 4 = complex tear, 5 = maceration). Articular cartilage was evaluated in six femoral and four acetabular subregions, with the labrum evaluated in four acetabular subregions. Our radiolologist has substantial-to-great interobserver agreement with another radiologist for articular cartilage and labrum outcomes (prevalence-adjusted and bias-adjusted kappa = 0.88 [95% CI 0.84 to 0.91] and 0.61 [95% CI 0.52 to 0.70] for cartilage and labral outcomes, respectively).

T1ρ and T2 mapping acquisitions were processed using in-house software developed in MATLAB (The MathWorks, Inc. Natick, MA). Cartilage T1ρ and T2 maps were generated by performing two-parameter fittings of single exponential decays with the Levenberg-Marquardt algorithm on a voxel-by-voxel basis according to the following equations:

1 $$\:S\left(TSL\right)={S}_{0}{e}^{-\frac{TSL}{{T}_{1\rho\:}}}$$

$$\:S\left(TE\right)={S}_{0}{e}^{-\frac{TE}{{T}_{2}}}$$

The first echo was not included in the T2 fitting procedure to reduce potential errors resulting from stimulated echoes in a multi-echo Carr–Purcell–Meiboom–Gill sequence^37,38^. Quality of the fittings was assessed with adjusted R^2^ maps.

**Fig. 2:**
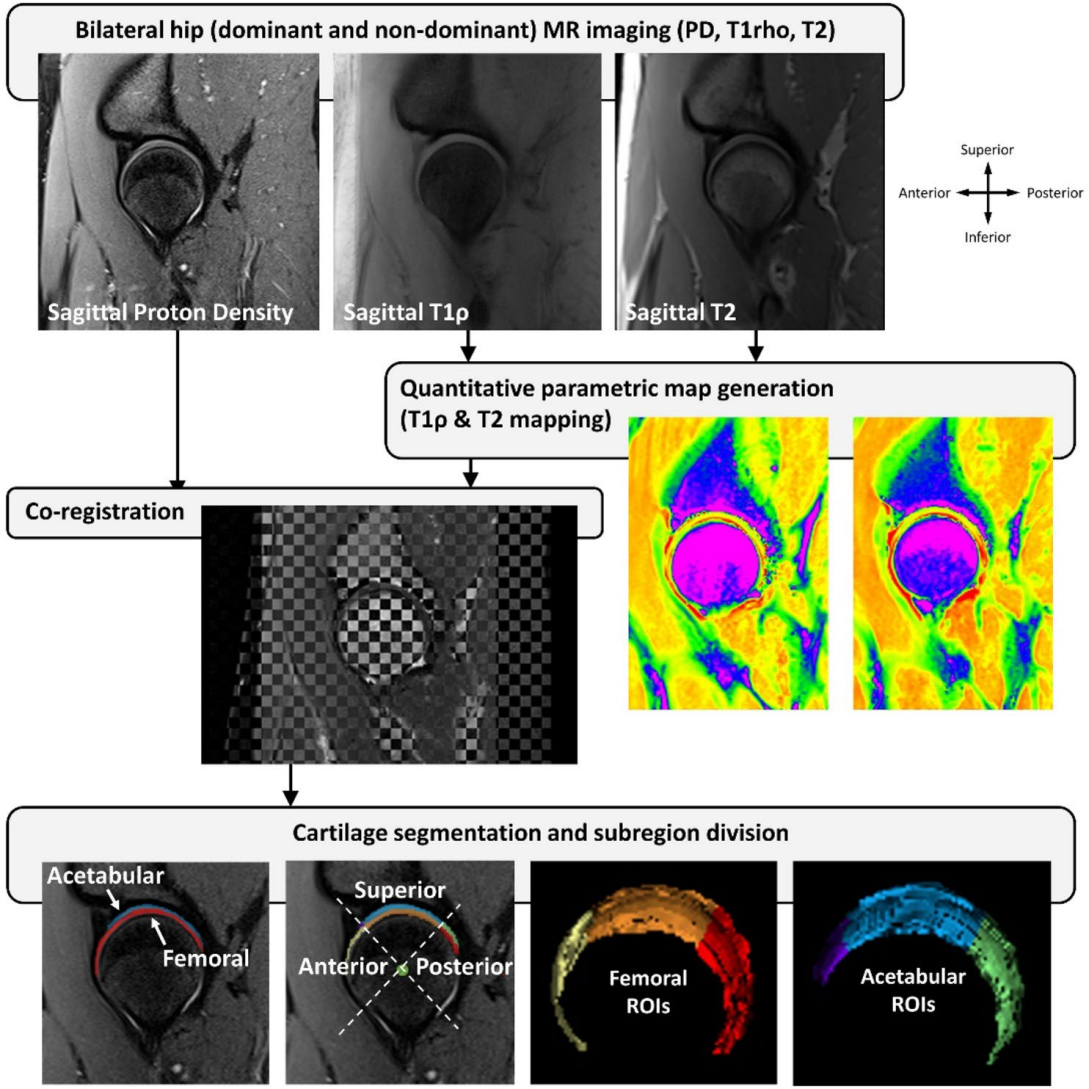
Assessment of cartilage composition on MRI scans. Cartilage T1ρ and T2 maps were generated and co-registered with the sagittal proton density image on which the femoral and acetabular cartilage compartments were segmented. T1ρ and T2 relaxation times were computed within the entire femoral and acetabular mask as well as the anterior, superior, and posterior subregions.

T1ρ and T2 maps were co-registered with the sagittal PD image on which the femoral and acetabular cartilage compartments were manually segmented using Analyze 14.0 (AnalyzeDirect, Overland Park, KS) to create a femoral and acetabular mask (Fig. 2). Segmentation was performed on a slice-by-slice basis spanning all slices. T1ρ and T2 relaxation times were computed as the average of all voxels within the segmented femoral and acetabular mask. Each cartilage mask was also subdivided into anterior, superior, and posterior subregions by utilizing a line connecting the centroids of the femoral head and neck (Fig. 2). Gluteus maximus, medius, and minimus were manually segmented on the Dixon sequence scan to acquire muscle volumes for both the Drive and Stride legs. All segmentations were reviewed by an expert rater (RKS) with 14 years of experience in musculoskeletal MRI analysis.

### Statistical analyses

Two-tailed analyses with α = 0.05 were performed with IBM SPSS Statistics (v29.0.1.0; IBM Corporation, Armonk, NY), unless otherwise described. Demographics between groups were compared using unpaired t-tests. Alpha angle at each clock position, acetabular version, and LCEA were analyzed using two-way one-repeated measure ANOVAs, with sport (Pitch1 vs. Run) and leg (Drive vs. Stride) as the between- and within-subject independent variables, respectively. Main effects for the independent variables were explored in the absence of a significant sport*leg interaction. The frequency of an alpha angle of ≥ 60°, acetabular version ≥ 20° and LCEA ≥ 40° were compared between sport and leg using Chi-square and McNemar’s tests, respectively. Paired t-tests were used to compare T2 and T1ρ relaxation times between the Drive and Stride legs in Pitch2. Relaxation times in the bilateral legs of Con were combined (due to the small sample size and to take advantage of regression to the mean) and compared to the Drive and Stride legs in Pitch2 via a one-way ANOVA with posthoc pairwise comparisons utilizing the false discovery rate approach of Benjamini and Hochberg (q < 0.05)^39^.

## Results

### Demographics

Pitch1 (*n* = 25) and Run (*n* = 13) were the same age (*p* = 0.78); however, Pitch1 were taller, heavier, and had a higher BMI than Run (all *p* < 0.001) (Table 1). Pitch1 had 6.5% (95% CI 4.8–8.2%) greater total hip aBMD in their stride leg compared to their drive leg (*p* < 0.001). There was no side-to-side difference in total hip aBMD in Run (0.6%; 95% CI − 0.5 to 1.7%).

**Table 1:** Participant characteristics.

Characteristic	CT participants		MRI participants	
	Pitch1	Run	Pitch2	Con
*n*	25	13	10	4
Age (year)	20.4 ± 1.4	20.6 ± 1.3	20.8 ± 1.0	21.6 ± 1.3
Height (m)	1.75 ± 0.06*	1.67 ± 0.06	1.74 ± 0.07^#^	1.63 ± 0.05
Mass (kg)	80.1 ± 10.6*	58.1 ± 6.7	78.7 ± 14.1	71.9 ± 10.3
Body mass index (kg/m^2^)	26.1 ± 3.3*	21.1 ± 1.1	25.8 ± 3.7	27.2 ± 5.4
Drive leg (R/L)^‡^	22/3	13/0	9/1	4/0
Age started playing (year)	6.8 ± 1.8	–	7.1 ± 1.6	–
Total years playing (year)	13.6 ± 2.2	–	13.7 ± 2.0	–

Data are mean ± SD, except for frequencies.

^‡^Drive leg is on the same side (i.e., ipsilateral) to the preferred throwing arm.

*p < 0.001 compared to Run.

^#^*p* < 0.001 compared to Con.

Pitch2 (*n* = 10) and Con (*n* = 4) were similar in age, body mass, and BMI (all *p* = 0.27 to 0.58); however, Pitch2 were taller than Con (*p* = 0.02) (Table 1). Pitch2 had 5.6% (95% CI 3.6–7.7%) greater total hip aBMD in Stride compared to Drive (*p* < 0.001).

### Hip morphology

**Fig. 3:**
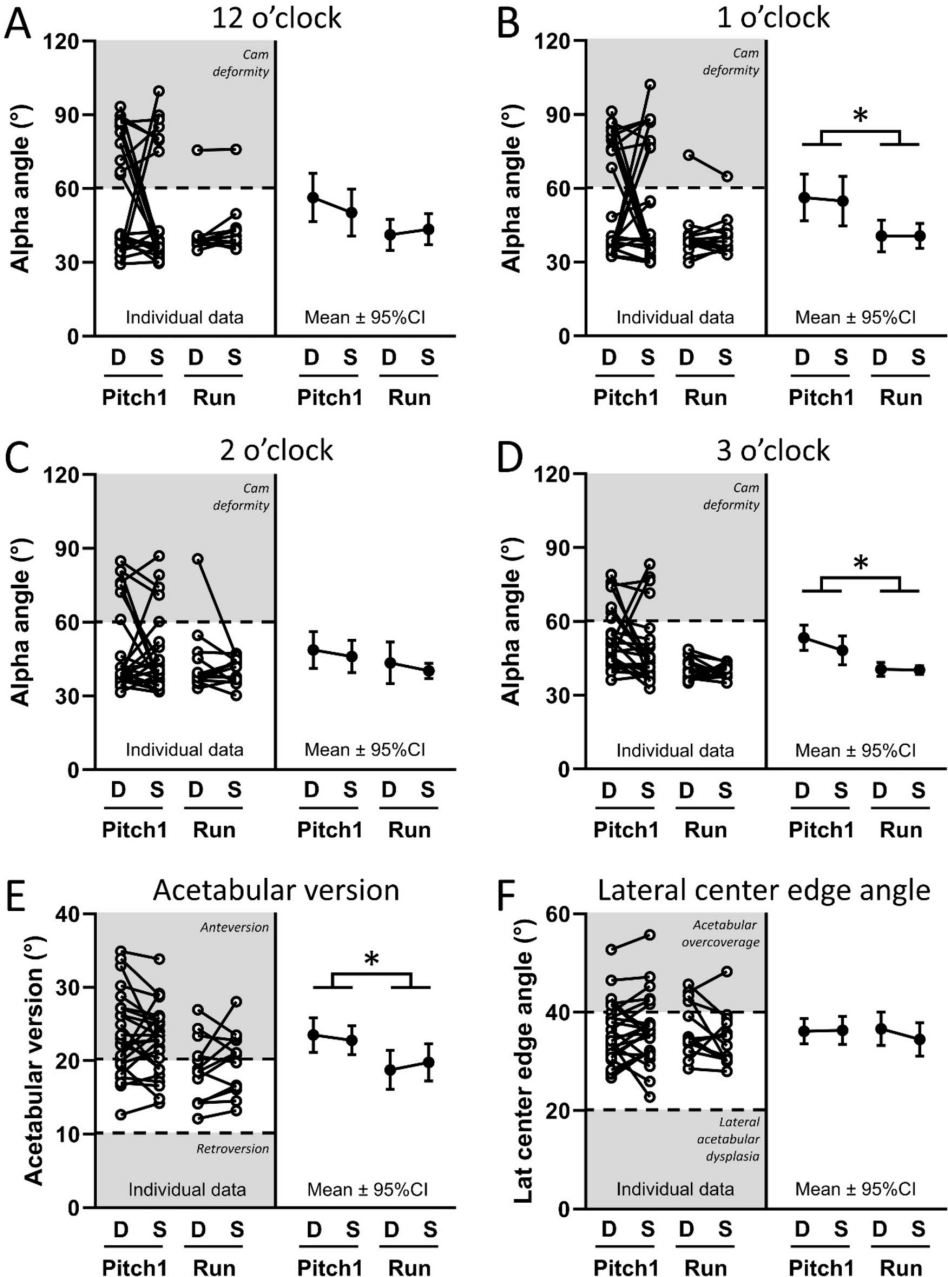
Alpha angle (°) in the proximal femur at the: (**A**) 12, (**B**) 1, (**C**) 2, and (**D**) 3 o’clock positions and (**E**) acetabular version and (**F**) lateral center edge angle in the drive (‘D’) and stride (‘S’) legs of softball pitchers (‘Pitch1’) and cross-country runners (‘Run’). The left side of each graph shows the paired data for each individual participant. The right side of each graph shows the group mean and 95% confidence interval (CI). *p < 0.05 for sport (Pitch1 vs. Run) main effect.

There was no interaction between sport (Pitch1 vs. Run) and leg (Drive vs. Stride) for alpha angle at any clock position (all *p* = 0.30 to 0.91, Fig. 3A-D), acetabular version (*p* = 0.17, Fig. 3E), or LCEA (*p* = 0.18, Fig. 3F). The maximum alpha angle across clock positions and legs differed between Pitch1 (73.5°; 95% CI 65.8° to 81.2°) and Run (48.3°; 95% CI 41.9° to 54.7°) (*p* < 0.001). Pitch1 had 36.6% (14.9°; 95% CI 4.3° to 25.5°) and 25.9% (10.5°; 95% CI 4.2° to 16.7°) greater alpha angle compared to Run at the 1 o’clock (Fig. 3B) and 3 o’clock (Fig. 3D) positions, respectively (all *p* < 0.01). Pitch1 had 20.1% (3.9°, 95% CI 0.7° to 7.1°) greater acetabular version than Run (*p* = 0.02, Fig. 3E). There was no main effect for sport on LCEA (*p* = 0.73, Fig. 3F) or main effect for leg on alpha angle at any clock position (all *p* = 0.24 to 0.88, Fig. 3A–D), acetabular version (*p* = 0.73, Fig. 3E), or LCEA (*p* = 0.26, Fig. 3F).

**Fig. 4:**
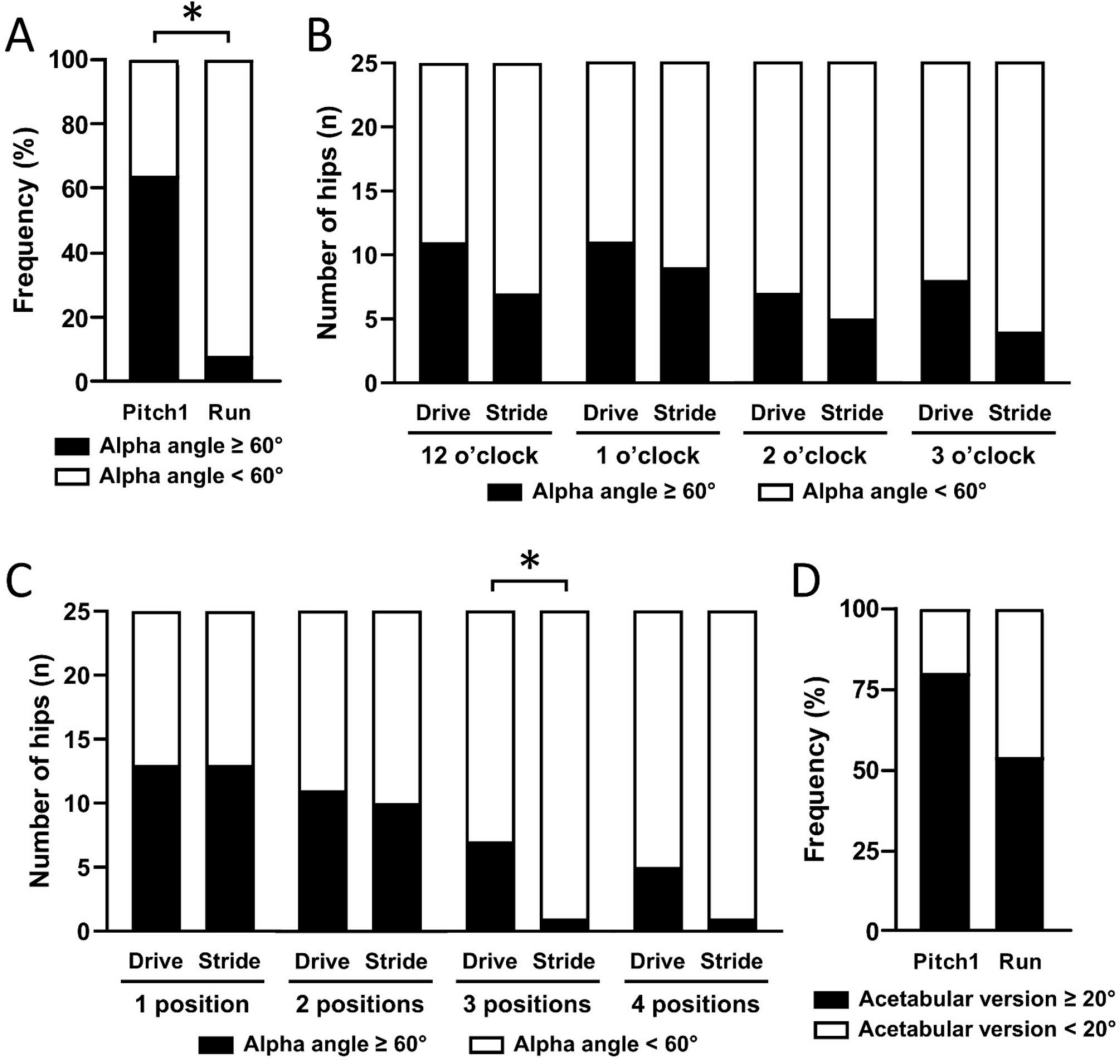
Frequencies of elevated alpha and acetabular version angles. (**A**) Frequency of alpha angle ≥60° in either leg of Pitch1 and Run. (**B**) Occurrence of an alpha angle ≥60° at each clock position in the Drive and Stride legs of Pitch1. (**C**) Occurrence of an alpha angle ≥60° at 1, 2, 3 or 4 clock positions in the Drive and Stride legs of Pitch1. (**D**) Frequency of acetabular version ≥20° in either leg of Pitch1 and Run. *p≤0.05.

Pitch1 (16-out-of-25 pitchers) were 21.3 (95% CI 2.4 to 192.0) times more likely to have an alpha angle ≥ 60° at one or more clock positions within either hip compared to Run (1-out-of-13 individuals) (*p* < 0.001, Fig. 4A). There was no Drive-to-Stride difference in the likelihood of having an alpha angle ≥ 60° at any of the clock positions in Pitch1 (all *p* = 0.29 to 0.75, Fig. 4B). Similarly, there were no Drive-to-Stride leg differences in the likelihood of having an alpha angle ≥ 60° at any one or two clock positions in Pitch1 (all *p* = 1.00, Fig. 4C). However, Drive more often had an alpha angle ≥ 60° at three clock positions compared to Stride in Pitch1 (*p* = 0.03). There was no difference between Pitch1 and Run in the frequency of acetabular version ≥ 20° (*p* = 0.09, Fig. 4D) or LCEA ≥ 40° (*p* = 0.75, *data not shown*) in either leg. There was no Drive-to-Stride difference in the likelihood of having acetabular version ≥ 20° or LCEA ≥ 40° in Pitch1 (all *p* = 1.00).

### Muscle morphology and cartilage health

Gluteus maximus and medius volumes were 3.1% (95% CI 0.3–5.9%) and 2.6% (95% CI 0.2–5.0%) larger in Stride compared to Drive (all *p* ≤ 0.04) (Supplementary file 2). There were no side-to-side differences in gluteus minimus volume (7.0%; 95% CI −0.5–14.4%) (*p* = 0.07) or differences in muscle volumes between Pitch2 and Con (all *p* = 0.15 to 0.64) (Supplementary File 2). In Pitch2, a simple (grade 2) labral tear was present in 6-out-of-10 pitchers and 7-out-of-20 hips (Drive, *n* = 2, Stride, *n* = 5; *p* = 0.58, McNemar’s test). Partial thickness cartilage loss was identified in four hips in Drive (at the superolateral acetabulum in two hips, posterior acetabulum in one hip, and anterior femoral head in one hip) and one hip in Stride had full thickness loss (at the anterior acetabulum).

T1ρ and T2 relaxation times were assessed in 18 hips (9 pitchers) and 20 hips (10 pitchers) in Pitch2, respectively. T1ρ was not assessed in one pitcher because of an MR scanner hardware issue. T2 relaxation times were assessed in 8 hips of Con. T1ρ relaxation times were not assessed in Con as a required software update removed the T1ρ sequence from the MR scanner prior to the scanning of Con.

There were no Drive-to-Stride differences in Pitch2 for T1ρ or T2 relaxation time in the total femoral or acetabular cartilage (all *p* = 0.31 to 0.95, Supplementary File 3). T2 relaxation times in the total femoral and acetabular cartilage did not differ between Pitch2 and Con (all *q* = 0.56 to 0.96, Supplementary File 3).

**Fig. 5:**
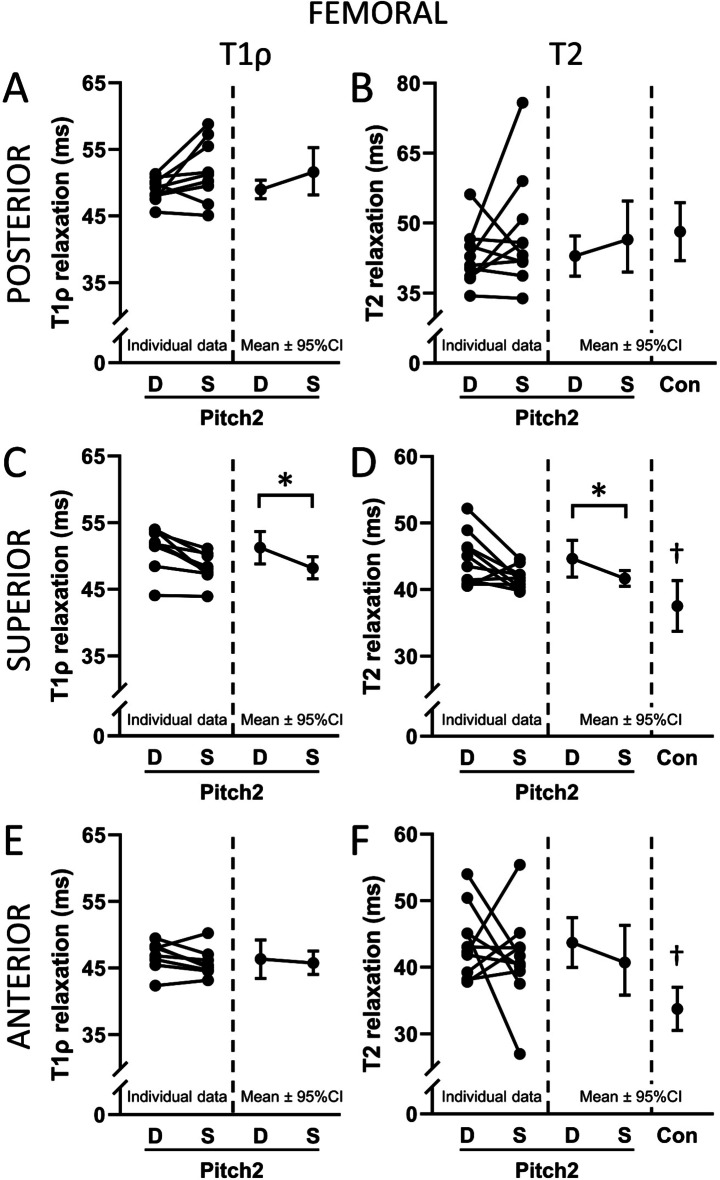
T1ρ (**A**,**C**,**E**) and T2 (**B**,**D**,**F**) relaxation times in the posterior, superior, and anterior femoral cartilage in the drive (‘D’) and stride (‘S’) legs of softball pitchers (‘Pitch2’) and T2 relaxation times in the legs of controls (‘Con’). The left side of each graph shows the paired data for each Pitch2 individual. The right side of each graph shows the group mean and 95% confidence interval (CI). *p <0.05 for Drive vs. Stride in Pitch2. ^†^*q* < 0.05 for Con vs. Drive and Con vs. Stride.

Drive in Pitch2 had 6.3% (3.0 ms; 95% CI 1.5 to 4.6 ms) and 7.1% (3.0 ms; 95% CI 0.3 to 5.6 ms) longer T1ρ and T2 relaxation times in the superior portion of the femoral cartilage compared to Stride, respectively (all *p* ≤ 0.03, Fig. 5C, D). T2 relaxation times in Pitch2 in the superior portion of the femoral cartilage of Drive and Stride were 18.9% (7.1 ms; 95% CI 3.9 to 10.3 ms) and 11.1% (4.2 ms; 95% CI 0.9 to 7.4 ms) longer than in Con, respectively (all *q* < 0.02, Fig. 5D). There were no Drive-to-Stride differences in Pitch2 for T1ρ or T2 relaxation times in the posterior (Fig. 5A, B) or anterior (Fig. 5E, F) portions of the femoral cartilage (all *p* = 0.07 to 0.60). T2 relaxation times in the anterior portion of the femoral cartilage of Drive and Stride in Pitch2 were 29.5% (10.0 ms; 95% CI 4.7 to 15.2 ms) and 22.3% (7.5 ms; 95% CI 2.3 to 12.8 ms) longer than in Con, respectively (all *q* < 0.01, Fig. 5F). T2 relaxation times in the posterior portion of the femoral cartilage of Drive and Stride in Pitch2 did not differ from Con (all *p* = 0.46 to 0.91, Fig. 5A, B).

**Fig. 6:**
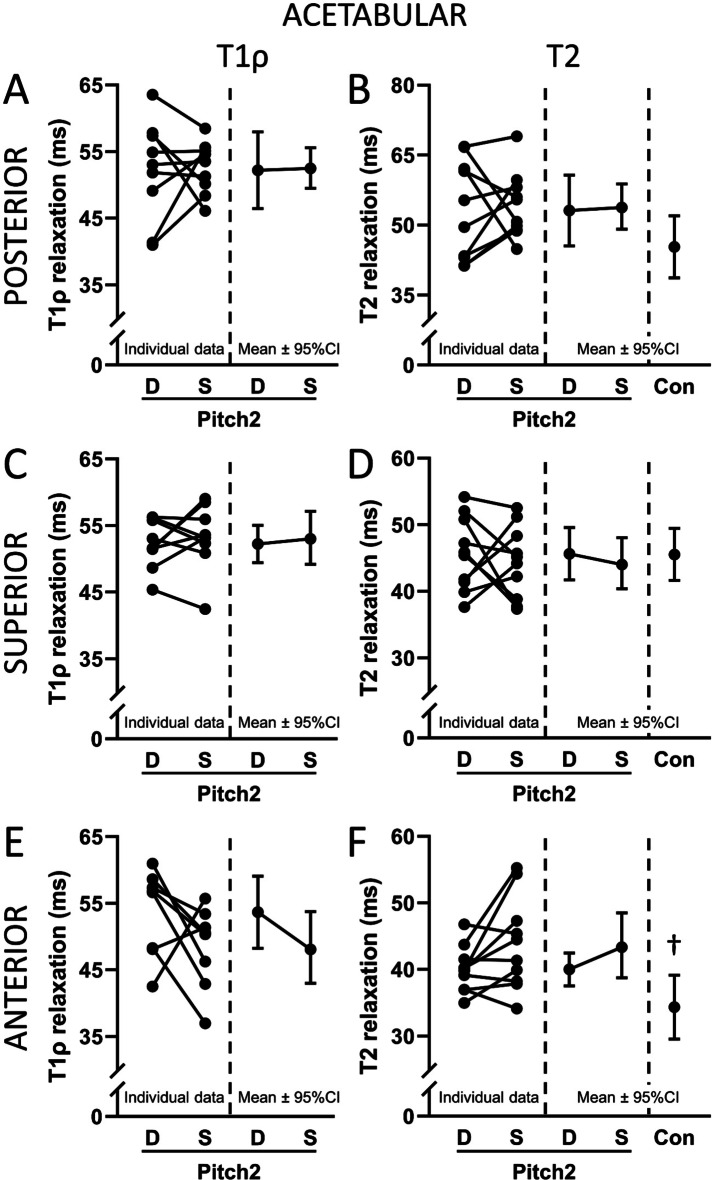
T1ρ (**A**,**C**,**E**) and T2 (**B**,**D**,**F**) relaxation times in the posterior, superior, and anterior acetabular cartilage in the drive (‘D’) and stride (‘S’) legs of softball pitchers (‘Pitch2’) and T2 relaxation times in the legs of controls (‘Con’). The left side of each graph shows the paired data for each Pitch2 individual. The right side of each graph shows the group mean and 95% confidence interval (CI). ^†^*q* < 0.05 for Con vs. Drive and Con vs. Stride.

There were no Drive-to-Stride differences in Pitch2 for T1ρ or T2 relaxation times in any regions of the acetabular cartilage (all *p* = 0.07 to 0.89) (Fig. 6). T2 relaxation times in Pitch2 in the anterior portion of the acetabular cartilage of Drive and Stride were 16.5% (5.7 ms; 95% CI 0.5 to 10.9 ms) and 27.7% (9.5 ms; 95% CI 4.3 to 14.7 ms) longer than in Con, respectively (all *q* < 0.05, Fig. 6E, F). T2 relaxation times in the posterior and superior portions of the acetabular cartilage of Drive and Stride in Pitch2 did not differ from Con (all *p* = 0.07 to 0.97, Fig. 6A-D).

## Discussion

In this study of female collegiate-level softball pitchers, we observed altered bony hip morphology and cartilage composition. 64% of pitchers had a cam morphology (alpha angle ≥ 60°) in at least one leg and 28% (7-out-of-25 pitchers) had bilateral cam morphology. In total, 52% of hips (26-out-of-50 hips) in pitchers had a cam morphology. When compared to collegiate-level cross-country runners, softball pitchers were 21.3 (95% CI 2.4 to 192.0) times more likely to have an alpha angle ≥ 60° within at least one hip. In terms of cartilage composition, softball pitchers had greater T2 relaxation times in the femoral cartilage compared to non-athletic controls and had side-to-side differences in T1ρ and T2 relaxation times in their femoral cartilage.

The cam morphology prevalence rate of 52% observed in softball pitcher hips is higher than the 19.1% reported in 598 hips of non-professional female athletes^40^. However, the latter study assessed alpha angle on a standard AP pelvic radiograph which can only detect cam morphologies at the single 12 o’clock/superior position and not the more anterior 1, 2, and 3 o’clock positions visible using the CT-based approach in the current study^41^. The cam morphology prevalence detected in our college-level softball pitchers appears comparable to the 48% assessed in female collegiate-level track & field, soccer, and volleyball athletes (61-out-of-126 hips)^42^ and female professional soccer players (19-out-of-40 hips)^43^. However, the prevalence rates in these previous studies are likely inflated relative to the current study.

Kapron et al.^42^ defined cam morphology by an alpha angle ≥ 50° and/or head-neck offset < 8 mm. Only 4.0% (5-out-of-126) of hips in their study reached the alpha angle threshold of ≥ 60° used in the current study. Similarly, Gerhardt et al.^43^, in their study of professional female soccer players, defined cam morphology by an alpha angle > 55° or the presence of subjectively identified features (e.g., excessive bone femoral head-neck junction, loss of femoral head sphericity, or femoral head-neck offset flatening). The use of a lower threshold and inclusion of hips based on other criteria increased prevalence, as indicated by the hips in their study with a positive cam morphology (*n* = 19) having an average alpha angle of only 60.8° (compared to 84.7° in cam morphology positive hips in the current study).

Cam morphology appears to develop prior to skeletal maturity in response to mechanical loading across the growth plate located at the femoral head-neck junction^44–46^. Softball pitchers in the current study started playing well prior to their adolescent growth period and they repetitively load their lower extremities, with vertical ground reaction forces equivalent to 1.4–1.9 times body weight being generated during each pitch^18,19,47^. Runners also repetitively load their lower extremities and with higher numbers of cycles and ground reaction forces relative to body weight than softball pitchers^48^. However, ground reaction forces only quantify the intensity of foot-ground impacts and not internal loads, with the latter being much greater due to muscle generated forces. Softball pitchers weighed 35% more than cross-country runners in the current study so absolute loads across the hip experienced by the pitchers (i.e., when not expressed relative to body mass) were likely much greater than in the runners. Albeit we do not know whether the mass of the pitchers differed from runners during adolescence when cam morphology develops. Softball pitchers also load their hips in positions and directions thought to be consequential with regards to the development of cam morphometry—namely, hip flexion, rotation and adduction^47,46^.

Pitchers in our study were asymptomatic, consistent with studies that show cam morphology is prevalent in asymptomatic populations. However, cam morphology is a risk factor for FAI syndrome^7^ and is associated with acetabular labral tears, chondral damage, and OA development^3,9–12^. In our second cohort of softball pitchers who underwent MRI (Pitch2 group), 7-out-of-20 hips were identified as having a labral tear and cartilage loss was evident in 5-out-of-20 hips. Whether the labral tears and cartilage loss are consequential in terms of future symptom development requires longitudinal surveillance.

Cam morphology may result in symptoms by potentiating impact between the femoral head-neck junction and acetabular rim. However, the extent of impact may be altered in softball pitchers due to altered acetabular morphometry. LCEA did not differ between pitchers and runners suggesting similar acetabular coverage; however, softball pitchers had 20% greater acetabular version angle, with 35-out-of-50 hips in pitchers having acetabular anteversion (version angle > 20°). There was no statistical difference in the frequency of acetabular anteversion between pitchers and runners (*p* = 0.09), but the greater mean acetabular version angle in pitchers may potentially delay impact between the femoral head-neck junction and acetabular rim. However, version angle explains only 4.7% of the variance in alpha angle in the current cohort of 50 hips in pitchers (*p* = 0.13, Pearson’s correlation coefficient) suggesting that those with the greatest alpha angle did not necessarily have corresponding increased acetabular version.

Assessment of hip cartilage composition in the second cohort of softball pitchers revealed altered composition. Compared to hips in control females of the same age, body mass, and BMI, the hips in softball pitchers had significantly longer T2 relaxation times in the superior subregion of the femoral cartilage and anterior subregion of both the femoral and acetabular cartilage. These observations suggest higher water content, lower glycosaminoglycan content and/or reduced integrity of the collagen network in the anterior and superior regions of softball pitcher’s hips. The anterior and anterosuperior subregions approximate the regions that cam morphology occurs and have been reported to have elevated relaxation times in the hips of individuals with FAI syndrome^30,31^. The changes observed in the softball pitchers may indicate early cartilage degeneration. Elevated T2 relaxation times at baseline in individuals with mild-to-moderate hip OA were associated with longitudinal progression of morphologic cartilage degeneration^49^.

Few studies have explored hip cartilage composition in athletes or in response to physical activity and joint loading activities^1^. Bittersohl et al.^50^ revealed T2* values consistent with cartilage degeneration in the hips of 20 elite rowers; however, less than half of the rowers were female and female specific data were not presented. Fernquest et al.^2^ observed elite adolescent male soccer players had a 4.85 ms greater acetabular cartilage T2 relaxation time than male controls and there was a positive correlation between physical activity level and relaxation time; however, no female athletes were included in the study. To our knowledge, the current study is the first report on hip cartilage composition in a cohort of female only athletes.

The softball pitchers in our study exhibited asymmetry between their drive (i.e., leg on the same side of the pitching arm) and stride legs. There was no asymmetry in alpha angle magnitude; however, drive legs had a higher prevalence of cam morphology across three clock positions than stride legs suggesting drive legs had a more extensive cam morphology. Drive legs also had significantly greater T1ρ and T2 relaxation times in the superior region of the femoral cartilage compared to the stride leg suggesting greater cartilage composition changes in the drive legs.

The greater changes observed in the drive leg are opposite to what we were anticipating as the stride leg is exposed to 35% greater vertical ground reaction forces during pitching^19^ and has greater proximal femur bone density and strength than the drive leg^20,21^. We confirmed the presence of greater proximal femur bone density in the stride leg in the current study and also found the stride leg had greater gluteus maximus and medius muscle volume, which corresponds with the higher use of these muscles during pitching compared to in the drive leg^47^. These cumulative data suggest that the stride leg is exposed to heightened mechanical loading during pitching compared to the drive leg. The observation of greater cartilage composition changes in the drive leg suggests the interaction with other factors which need further investigation and elucidation.

Strengths of our study include the investigation of a female-only population, use of a within-subject controlled model, study of both morphological and cartilage composition outcomes, and inclusion of comparison groups. However, the study also possesses limitations. The study was cross-sectional with a small sample size, we did not assess the reproducibility of our outcomes, and we were unable to assess T1ρ relaxation times in control hips as the sequence was removed from the scanner during a required software update. Also, MRI relaxation times at the spherical femoral head are vulnerable to magic angle effects which may have influenced outcomes away from the most central slices.

In summary, the current exploratory study provides evidence that softball pitchers exhibit different bony hip morphology and cartilage composition. Both hips were affected, with greater changes in cartilage composition in the drive leg compared to the stride leg. Any long-term consequences of the observed structural and composition phenotypes in terms of symptoms and the development of osteoarthritis remain unknown and require further investigation.

## Electronic supplementary material

Below is the link to the electronic supplementary material.

Supplementary Material 1

## Data Availability

The data generated during the current study are available from the corresponding author on reasonable request.
