# Supplementary material for: Asymptomatic female softball pitchers have altered hip morphology and cartilage composition
Source: Sci Rep. 2025 Jan 25;15:3262. doi: 10.1038/s41598-025-87839-2 (PMC11762768; doi:10.1038/s41598-025-87839-2)

Supplementary file 1. MR imaging protocol and parameters utilized

Imaging parameter	Sagittal PD	Sagittal PD SPAIR	Coronal PD SPAIR	Axial PD SPAIR	Sagittal T1ρ	Sagittal T2	Axial Dixon
Repetition time (msec)	2880	3950	3850	3850	6.3	2240	3.8
Echo time/s (msec)	19	19	34	34	-	10.4, 20.8, 31.2, 41.6, 52.0, 62.4	2.46
Spin-lock times (msec)	-	-	-	-	0, 10, 20, 30, 40	-	-
Matrix	256x256	256x256	256x256	256x256	256x256	192x192	288 x 288
Field of view (mm)	170x170	170x170	170x170	170x170	160 x 160	180 x 180	380 x 380
In-plane resolution (mm)	0.7 x 0.7	0.7 x 0.7	0.7 x 0.7	0.7 x 0.7	0.6 x 0.6	0.9 x 0.9	1.3 x 1.3
Slice thickness (mm)	2	2.5	2.5	2.5	3	2.5	3
Distance factor	65%	40%	60%	60%	20%	100%	20%
Gap between slices (mm)	1.3	1	1.5	1.5	0.6	2.5	0.6
Flip angle (°)	130	130	140	140	10	180	9
Number of slices	32	35	28	28	32	22	96
Bandwidth/Pixel (Hz/pixel)	391	391	80	80	400	248	1020
Acquisition time (mins)	2:00	2:47	5:12	5:12	11:54	8:26	0:27

PD = proton density; SPAIR = spectral attenuated inversion recovery

Supplementary file 2. Contouring (A) and 3D reconstruction (B) of the gluteus minimus, medius, and maximus muscles in the drive ('D') and stride ('S') legs. Gluteus maximus (C), medius (D), and minimus (E) volume in the D and S legs of softball pitchers ('Pitch2') and controls ('Con'). The left side of each graph shows the paired data for each Pitch2 individual. The right side of each graph shows the group mean and 95% confidence intervals (CI) for both Pitch 2 and Con.

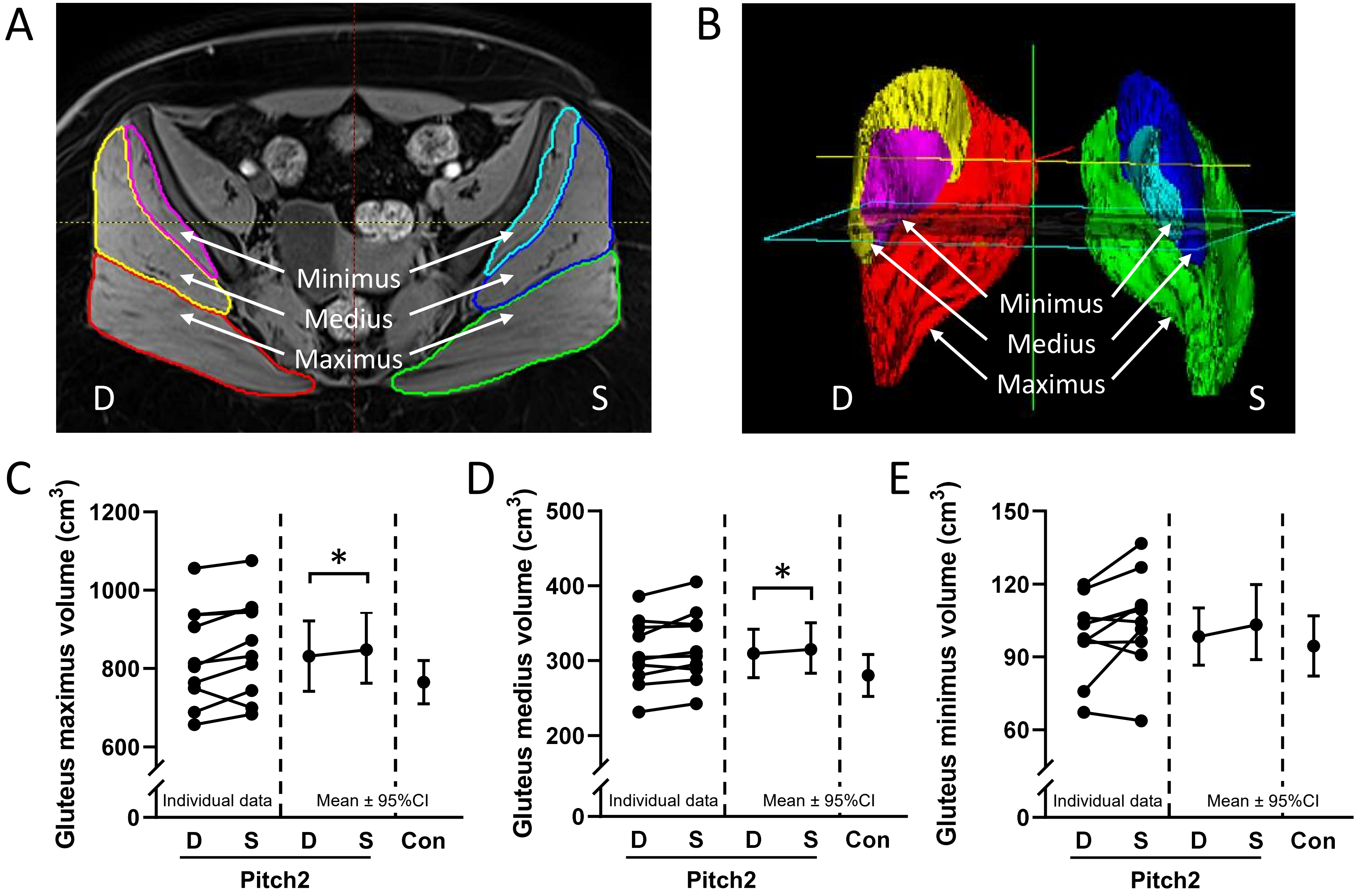

Supplementary file 3. T1ρ (A,C) and T2 (B,D) relaxation times in the total femoral and acetabular cartilage in the drive ('D') and stride ('S') legs of softball pitchers ('Pitch2') and T2 relaxation times in the legs of controls ('Con'). The left side of each graph shows the paired data for each Pitch2 individual. The right side of each graph shows the group mean and 95% confidence interval (CI).

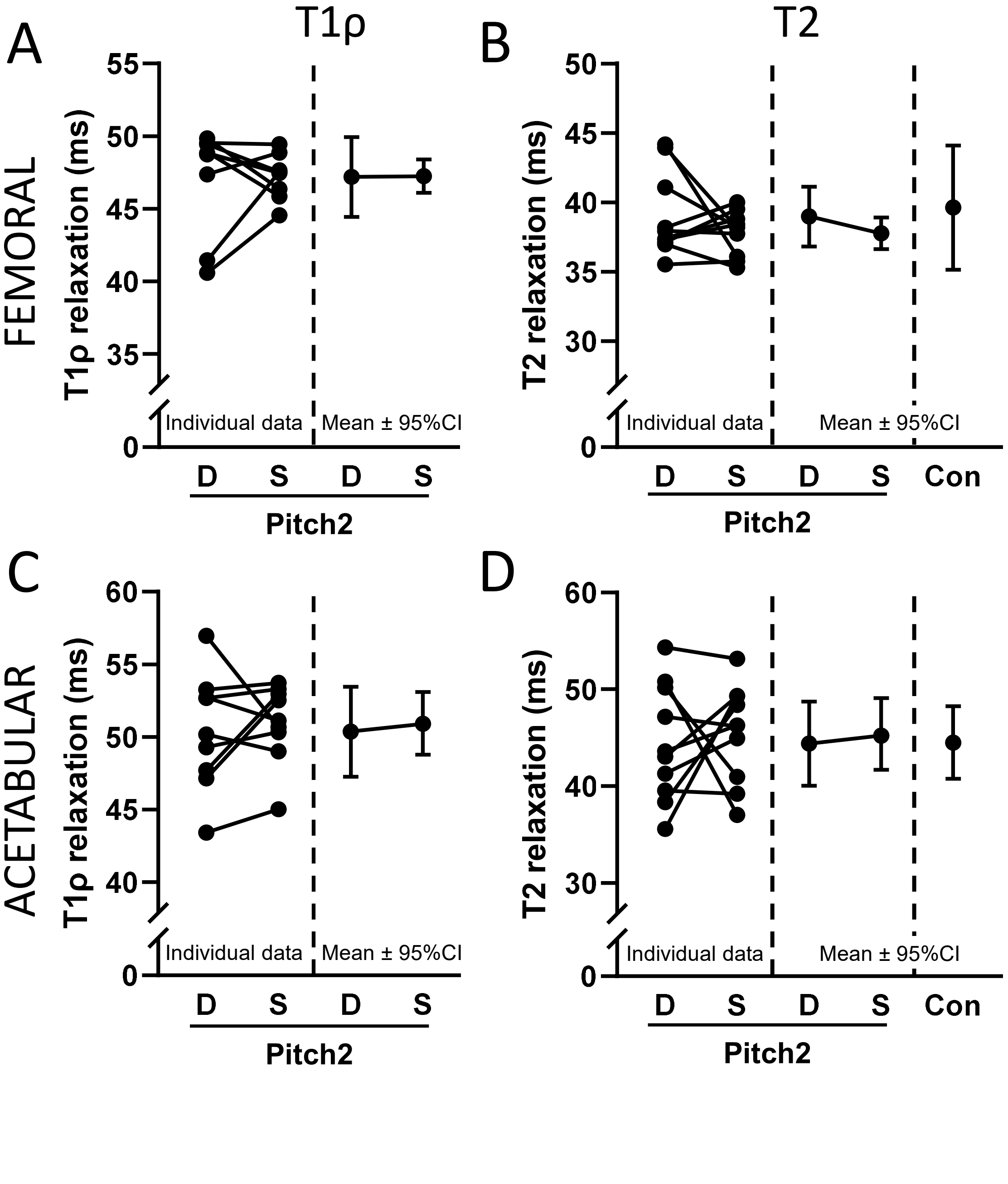

Supplement: Supplementary file 1 — Supplementary Material 1 [file 41598_2025_87839_MOESM1_ESM.pdf]
